# Diagnostic test accuracy: application and practice using R software

**DOI:** 10.4178/epih.e2019007

**Published:** 2019-03-28

**Authors:** Sung Ryul Shim, Seong-Jang Kim, Jonghoo Lee

**Affiliations:** 1 Department of Preventive Medicine, Korea University College of Medicine, Seoul, Korea; 2 Urological Biomedicine Research Institute, Soonchunhyang University Hospital, Seoul, Korea; 3 Department of Nuclear Medicine, Pusan National University Yangsan Hospital, Pusan National University School of Medicine, Yangsan, Korea; 4 BioMedical Research Institute for Convergence of Biomedical Science and Technology, Pusan National University Yangsan Hospital, Yangsan, Korea; 5 Department of Internal Medicine, Jeju National University Hospital, Jeju National University School of Medicine, Jeju, Korea

**Keywords:** Meta-analysis, Diagnostic test accuracy, Receiver-operating characteristic curve, Likelihood ratios, Mada, Reitsma

## Abstract

The objective of this paper is to describe general approaches of diagnostic test accuracy (DTA) that are available for the quantitative synthesis of data using R software. We conduct a DTA that summarizes statistics for univariate analysis and bivariate analysis. The package commands of R software were “metaprop” and “metabin” for sensitivity, specificity, and diagnostic odds ratio; forest for forest plot; reitsma of “mada” for a summarized receiver-operating characteristic (ROC) curve; and “metareg” for meta-regression analysis. The estimated total effect sizes, test for heterogeneity and moderator effect, and a summarized ROC curve are reported using R software. In particular, we focus on how to calculate the effect sizes of target studies in DTA. This study focuses on the practical methods of DTA rather than theoretical concepts for researchers whose fields of study were non-statistics related. By performing this study, we hope that many researchers will use R software to determine the DTA more easily, and that there will be greater interest in related research.

## INTRODUCTION

General pairwise meta-analysis calculates the effect size, such as relative risk and odds ratio (OR) for binary data and the mean difference for continuous data. By contrast, the diagnostic test accuracy (DTA) simultaneously combines two effect sizes, such as the sensitivity and specificity or positive predictive value and negative predictive value [1-3].

Therefore, DTA is more complex than pairwise meta-analysis, which has one result value. The expansion to multivariate analysis with more than two results inevitably leads to the introduction of the multi-layer concept, which requires some degree of mathematical understanding as well as an ability to use statistical programs.

This study focuses on the procedures involved in running the R software (Supplementary Material 1) as well as the concepts of producing summary statistics, which need to be understood for DTA.

In this study, the previous meta-analysis studies performed by the authors [1-3] are reviewed using R software. Furthermore, this study requires prior knowledge about the meta-analysis of diagnostic tests because it first deals with the types and changes of the effect size to calculate the summary statistics for DTA.

## UNDERSTANDING DIAGNOSTIC TEST ACCURACY

**Figure 1.**
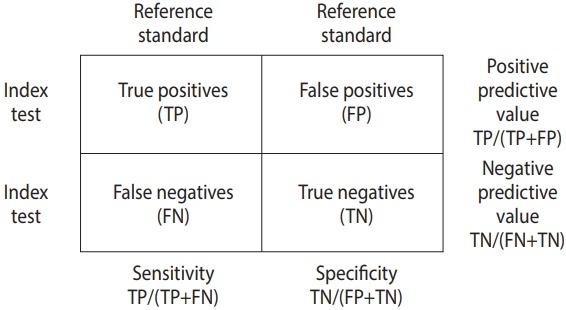
Summary statistics for diagnostic test accuracy.

The data for DTA assumes a 2×2 table form in which the row cells are distinguished by the presence or absence of a test, and the column cells are distinguished by the presence or absence of a disease (Figure 1).

### Summary statistics for diagnostic test accuracy

The DTA is represented by the summary statistics and summary line from four sets of basic data, namely true positive (TP), false positive (FP), false negative (FN), and true negative (TN). Representative summary statistics are the sensitivity, specificity, diagnostic odds ratio (DOR), and forest plot, and an example summary curve is the summary receiver operating characteristic (SROC) curve (Table 1).

### Diagnostic test accuracy model

To calculate the summary statistics for the DTA, an appropriate model should be selected, as with pairwise meta-analysis. Models that simultaneously consider the sensitivity and specificity include the Moses–Littenberg SROC model [4,5], bivariate model [6], and hierarchical SROC (HSROC) model [7].

The Moses–Littenberg model is a simple model that was created in the early stage to determine the DTA, and it estimates the SROC using simple linear regression. This is similar to the fixed-effect model in pairwise meta-analysis, and cannot estimate the heterogeneity between studies. Furthermore, this model cannot distinguish between within-study and between-study variations in all variations, and can perform limited analysis because it only provides the SORC curve without parameter estimates, standard deviation, or confidence intervals (CIs).

To overcome the disadvantages of the Moses–Littenberg model, the bivariate model and HSROC model were developed based on the hierarchical model. These two models provide the same value mathematically when there is no covariate [8,9]. This is similar to the random-effect model in pairwise meta-analysis. Both models can estimate the within-study and between-study variation of studies, that is, the heterogeneity.

The bivariate model assumes a binominal distribution that directly models the sensitivity and specificity for within-study variations, while assuming a bivariate normal distribution for between-study variation. However, the HSROC model assumes a binominal distribution for within-study variations, while assuming a hierarchical distribution for parameters included in the logistic model by applying the logistic regression model to determine the probability of a binominal distribution for between-study variation.

The R “mada” package reitsma model, which we will practice in this book, calculates the summary statistics and estimates the SROC curve using the bivariate model by default.

### Calculation of effect size

Examine the sensitivity and specificity in Table 1.

The sensitivity is TP/(TP+FN), and the specificity is TN/(TN+FP), which are proportion-type data.

For these proportion-type data, logit-transformed data are used more often than raw data. The logit transformation is a method of adjusting the data distribution according to statistical assumptions. The proportion-type data are limited between the lower and upper limits of 0 and 1, respectively. To convert these data to make them appropriate for the assumptions of statistics, their upper and lower limits should be released by performing multiplication and log transformations, respectively. This is called logit transformation.

Upon completion of the calculation of the summary statistics for DTA, they are reverted to their original values for interpretation. In the practice using R, we will calculate the logit-transformed sensitivity and specificity using the “metaprop” function of the “meta” package and then revert them to their original values to interpret them. Thus, we should understand why the effect size is transformed.

## DIAGNOSTIC TEST ACCURACY USING THE “mada” AND “meta” PACKAGES OF R

**Figure 2.**
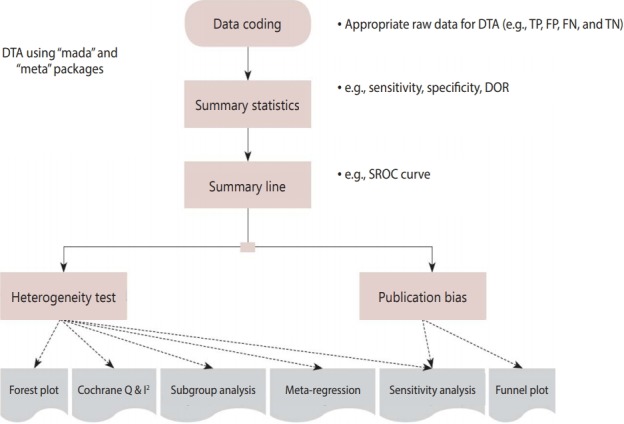
Flow chart of diagnostic test accuracy (DTA) using R “mada” & “meta” package. TP, true positive; FP, false positive; FN, false negative; TN, true negative; DOR, diagnostic odds ratio; SROC, summary receiver operating characteristic.

Figure 2 shows the flow of the DTA. First, when coding the data, we must change the variable name appropriately for the corresponding function. After selecting a meta-analysis model (fixed or random), the total effect size is presented, the heterogeneity is verified, and the publication bias is then verified and reported.

The “mada” package is required to analyze the DTA in R. After “mada” is installed, you will be promoted to install “mvtnorm,” “ellipse,” and “mvmeta.” Thus, you should install them in advance as follows:

·install.packages(“mada”)

·install.packages(“mvtnorm”)

·install.packages(“ellipse”)

·install.packages(“mvmeta”)

In addition, you should install the “meta,” “metafor,” and “rmeta” packages for general pairwise intervention meta-analysis in R as follows:

·install.packages(“meta”)

·install.packages(“metafor”)

·install.packaqes(“rmeta”)

The main explanations are applicable to the “mada” and “meta” packages. For detailed explanations about the “mada” package, refer to detailed codes, documents, and references for the package [10].

We mark R commands with a dot (‘·’) in front of them, to distinguish them from the main text. When long commands are extended to the next line, there is no dot at the beginning of the next line. Thus, when you enter the command in the R software, you must type them without the dot (‘·’) in front of them.

### Data coding and loading

As an example of the DTA, the urine sample measuring the albumin concentration method (Table 2) was selected from among the test methods for microalbuminuria in diabetes patients [2,3,11]. Subgroup (g) 1 consists of Western European counties, and 0 consists of countries other than Western European countries.

Load the example file from the working folder with the following command. Note that R prefers comma-separated values (csv) file format. Thus, you should save Table 2 as “dta_shim.csv” in the specified working folder.

·dta_shim <- read.csv (“dta_shim.csv”, header=TRUE)

read.csv is a function for loading a csv file. The above command means to load the file “dta_shim.csv” and use the first variable name (header=TRUE). This loaded file is saved as “dta_shim” in the R memory. To confirm this, enter the specified data in the View() function.

### Summary statistics

The “mada” package, which is a bivariate model for calculating the summary statistics for the DTA, does not provide the total effect sizes of summary statistics (sensitivity, specificity, and DOR) and only provides the effect size of individual studies as a forest plot, which is inconvenient.

Therefore, it is more natural to check the value of each summary statistic by performing univariate analysis using the “meta” package first, and then to present an SROC curve using the “mada” package.

### Univariate analysis

Calculate the sensitivity, specificity, and DOR and plot them using the univariate analysis model.

Load the meta package to perform meta-analysis:

·library(meta)

#### Sensitivity

The “meta” package includes many functions. Among them, the “metaprop” function calculates the total effect size using the number of events (event) and the number of samples (n) from proportion-type data.

· sensitivity_logit <- metaprop(dta_shim$TP, dta_shim$TP+ dta_shim$FN, comb.fixed=FALSE, comb.random=TRUE, sm=“PLOGIT”, method.ci=“CP”, studlab=dta_shim$id, byvar=dta_shim$g)

·print(sensitivity_logit, digits=3)

In sensitivity analysis, the number of events is TP and the number of samples is TP+FN. The variables of these data in R can be indicated by using the symbol ‘$’ (for example, write “dta_shim$TP” to indicate the TP variable of the dta_shim data). After sequentially entering the number of events (dta_shim$TP) and the number of samples (dta_shim$TP+dta_shim$FN) in the metaprop function, input other optional arguments at the end.

To calculate the effect size from proportion-type data, the method of reverting after logit transformation was used. Besides, you can enter sm=“PRAW” to use the raw data without transformation, or sm=“PLN” to find the reverted value after log transformation.

For consistency with the assumptions of the statistic model, and to consider the symmetricity and distribution of data, it is desirable to transform proportion-type data (log transformation or logit transformation) as they produce conservative results. However, many previous studies and statistical models have broadened the operation scope of researchers. Thus, it is necessary to find and use an appropriate method for the research results.

Even if data transformation is performed, the “metaprop” function automatically reverts and shows the total effect size that can be interpreted.

Furthermore, there are several methods for calculating the confidence interval, but the default Clopper–Pearson method is recommended as it is not too complex (method.ci=“CP”).

The random effect model was used, and comb.fixed=FALSE and comb.random=TRUE are also entered. The desired model can be selected by using FALSE or TRUE.

**Figure 3.**
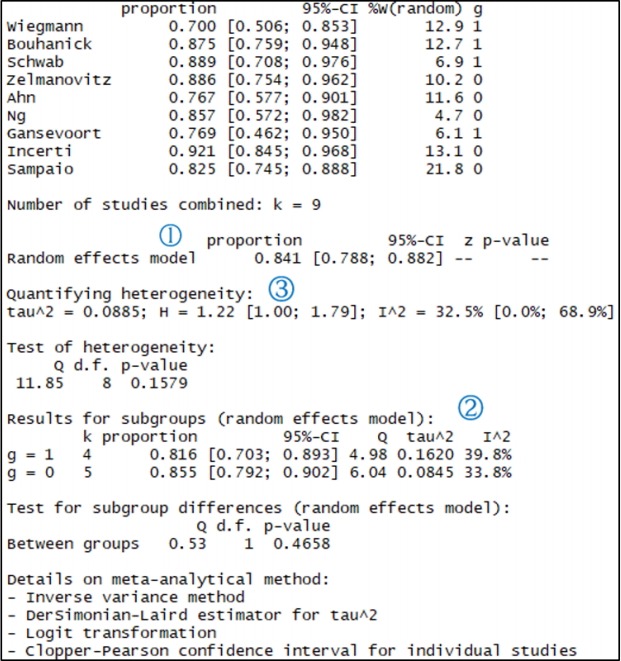
Univariate analysis: sensitivity. CI, confidence interval; g, subgroup.

Studlab=study indicates the name of individual studies. To show the result by subgroup, enter “byvar=g” where g is the variable name representing the subgroup. The results obtained when using the “metaprop” function are assigned to sensitivity_logit, and the result is shown in Figure 3.

The results from sensitivity_logit in Figure 3 are examined below one-by-one.

① Shows the total effect size of all nine studies. The proportion of the random effect model was 0.841 (95% CI, 0.788 to 0.882).

② Shows the result corresponding to the subgroup. The random model shows slight differences in sensitivity according to the subgroup (0 vs. 1). Based on the random effect model, the proportion is 0.816 for Western Europe countries and 0.855 for other countries. These values need to be tested using meta regression analysis according to country group later.

③ Shows the heterogeneity of all studies. The Higgins’ I^2^ of the heterogeneity is determined by subtracting the number of degrees of freedom from the Cochrane Q statistics, and then again dividing the resulting value by the Cochrane Q statistics. Thus, it quantifies the heterogeneity in a consistent manner. Values between 0% and 40% indicate that the heterogeneity may not be important; values between 30% and 60% indicate moderate heterogeneity; values between 50% and 90% indicate substantial heterogeneity; and values between 75% and 100% indicate considerable heterogeneity. The p-value of the Cochrane Q statistics is 0.1, which is a somewhat wide range of significance [3].

In this sensitivity analysis, the Higgins’ I^2^ is 32.5% and the Cochrane Q statistics p-value is 0.158, which suggest weak heterogeneity.

In addition, the calculation process for the results is revealed at the bottom of Figure 3. The inverse variance method is a basic meta-analysis method, and uses the inverse variance of the relevant study when calculating the weights of individual studies. The DerSimonian-Laird estimator indicates that the tau value was used when calculating the between-study variance.

Furthermore, logit transformation and Clopper–Pearson method were used.

■ Forest plot
· forest(sensitivity_logit, digits=3, rightcols=c(“effect”, “ci”), xlab=“Sensitivity”)

Enter the corresponding meta-analysis model (sensitivity_logit) in the forest function. Then, various options can be entered to facilitate identification. “digits=3” indicates that it shows only down to three decimal places, and “rightcols=c(“effect,” “ci”))” indicates that it shows the effect size and CI while omitting only the weight at the right side of the forest plot.

In addition, the addition of colors or the addition/removal of certain information is possible at one’s discretion. You can learn more details by practicing the meta package.

The forest plot provides the same information as the above-mentioned total effect size. Furthermore, within-study and between-study variation can be easily identified by the graphic representation of the effect size of individual studies.

For example, it can be seen that Gansevoort, Ng, Wiegmann, and Ahn have large within-study variations, and Wiegmann and Incerti have large between-study variations.

#### Specificity

· specificity_logit <- metaprop(dta_shim$TN, dta_shim$TN+ dta_shim$FP, comb.fixed=FALSE, comb.random=TRUE, sm=“PLOGIT”, method.ci=“CP”, studlab=dta_shim$id, byvar=dta_shim$g)
·print(specificity_logit, digits=3)

In the specificity analysis, the number of events is TN and the number of samples is TN+FP. After sequentially entering the number of events (dta_shim$TN) and the number of samples (dta_shim$TN+dta_shim$FP) in the metaprop function, input other optional arguments, respectively. The explanation after this is identical to that of the sensitivity analysis.

We will examine the results of specificity_logit one-by-one.

The total effect size of all nine studies is shown. The proportion of the random effect model was 0.861 (95% CI, 0.794 to 0.909).

The random model shows almost no difference in terms of the effect size between the subgroup (0 vs. 1). The Higgins’ I^2^ in this specificity analysis is 78.3%, and the p-value of Cochrane Q statistics is <0.0001, which indicates the existence of heterogeneity.

■ Forest plot
· forest(specificity_logit, digits=3, rightcols=c(“effect”, “ci”), xlab=“Specificity”)

The explanation for this command is the same as that for the sensitivity analysis.

#### Diagnostic odds ratio

The “meta” package includes several functions. Among them, the “metabin” function calculates the total effect size from binary data when there exist all of the raw data. The respective sensitivity and specificity are proportion-type data, but the DOR of the 2×2 format is binary data.

· DOR_model <- metabin(TP,TP+FP,FN,FN+TN, sm=”OR”, comb.fixed=FALSE,comb.random=TRUE, method=“Inverse,” id, byvar=g, data=dta_shim)

·print(DOR_model)

For binary data, enter TP, TP+FP, FN, and FN+TN in this order.

Write OR for effect size (sm=“OR”) and use the general inverse variance method for weights of individual studies (method=“Inverse”).

To set the random effect model considering the between-study variations, additionally enter “comb.fixed=FALSE” and “comb.random=TRUE.”

“id” indicates the name of the individual study, and “data=dta_shim” specifies the data “dta_shim” loaded to the R memory. To show the result for each g, enter “byvar=g,” where g is the variable name for the g. The results of the metabin function are assigned to the DOR model.

■ Forest plot
· forest(DOR_model, digits=3, rightcols=c(“effect”, “ci”), xlab =“Diagnostic Odds Ratio”)

**Figure 4.**
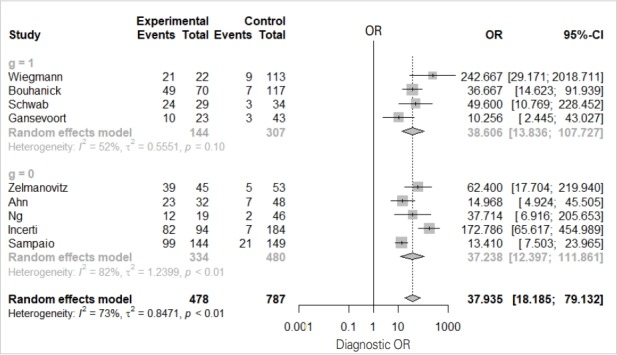
Univariate analysis: diagnostic odds ratio. OR, odds ratio; CI, confidence interval; g, subgroup.

We will examine the results of the DOR_model in Figure 4 one-by-one.

The total effect sizes of all nine studies are shown. The OR of the random effect model is 37.935 (95% CI, 18.186 to 79.132) and p-value <0.0001. In this diagnosis test, the OR for the positive result among persons with a disease was approximately 38 times higher than the OR for positive results among persons with no disease.

It appears that the random model has almost no difference according to subgroup (0 vs. 1).

The Higgins’ I^2^ of all studies is 72.7%, and the p-value of the Cochrane Q statistics is 0.0003, indicating that there is heterogeneity.

### Bivariate analysis

The “mada” package for bivariate analysis does not directly present the sensitivity, specificity, and DOR as in Meta-DiSc or STATA, which are other DTA applications. Thus, to show the combined overall statistics with “mada” package you should check the source code and calculate it manually.

Therefore, in this study, the summary statistics were analyzed separately for sensitivity, specificity, and DOR by performing univariate analysis. In the following bivariate analysis, only the SROC curve is estimated using the “mada” package.

Before loading the “mada” package, the “meta” package that was used before should be unloaded, because “mada” and “meta” both use the “forest” function, which may not be executed if it is called simultaneously by both packages.

·detach(package:meta)

#### Diagnostic test accuracy summary line (summary receiver operating characteristic curve)

Load the “mada” package for bivariate analysis:

·library(mada)

To see the forest plots of univariate analysis for sensitivity, specificity, and DOR using the “mada” package, enter the following commands:

· forest(madad(dta_shim), type=“sens”, xlab=“Sensitivity”, snames=dta_shim$id)

· forest(madad(dta_shim), type=“spec”, xlab=“Specificity”, snames=dta_shim$id)

·forest(madauni(dta_shim))

These plots are the same as those obtained in the univariate analysis, and are not recommended because they do not show the overall effect size of the summary statistics.

In the “mada” package, use the reitsma function, which is appropriate for a bivariate model.

·fit <- reitsma(dta_shim, correction.control=“single”)

·summary(fit)

Enter the dta_shim data in the reitsma function. It becomes impossible to calculate if there is ‘0’ in a data cell. To prevent this, you can enter 0.5 in all cells of every study (correction.control= “all”), or correct only the cell of the corresponding study (horizontal) (correction.control=“single”). In the options, you can adjust it to a random value such as ‘correction=0.5,’ where 0.5 is the default value. For models using the reitsma function, ‘fit’ is assigned.

In addition, you can refer to the area under the curve (AUC), which is 0.906, in the middle of the console window and the values corresponding to the HSROC model.

**Figure 5.**
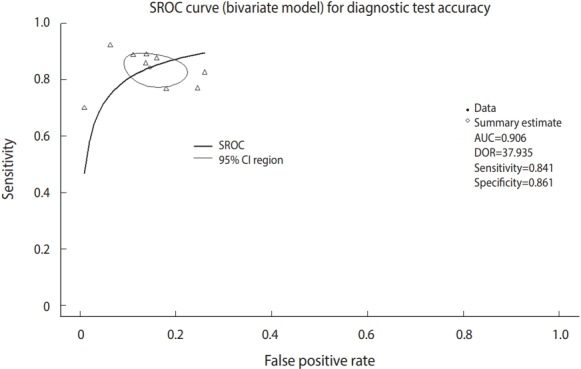
Summary receiver operating characteristic (SROC) curve (bivariate model) for diagnostic test accuracy. CI, confidence interval; AUC, area under the curve; DOR, diagnostic odds ratio.

Now, we will draw the SROC curve (Figure 5). The graphs will be drawn in the order of commands by overlapping because the first SROC curve remains in the memory.

· plot(fit, sroclwd=2, xlim=c(0,1), ylim=c(0,1), main=“SROC curve (bivariate model) for Diagnostic Test Accuracy”)

“plot” is a graph drawing function. Enter the set model fit. “sroclwd=2” indicates the thickness of the SROC curve. Adjust the units of the x and y axes by adjusting xlim and ylim, respectively. The current graph shows the range from a minimum of 0 to a maximum of 1.

·points(fpr(dta_shim), sens(dta_shim), pch=2)

Enter the individual study in points. fpr() and sens() respectively indicate the false positive rate and sensitivity of individual studies in the corresponding data. pch=2 indicates a triangle shape. You can choose from among various shapes: rectangle (0), circle (1), triangle (2), cross (3), scissors (4), rhombus (5), inverted triangle (6), star (8), and black dot (20). The black dot (20) appears to have the best discrimination (Figure 5).

·legend(“bottomleft,” c(“SROC,” “95% CI region”), lwd=c(2,1))

There is an annotation for each curve at the left bottom of the SROC curve.

### Heterogeneity review

Once the summary statistics and the SROC summary line are presented, we have the major components of the DTA. Then, if there is any significant heterogeneity of study, researchers should verify it and report the heterogeneity factors. The basic assumption of the SROC curve is that the shape of the ROC curve is identical in all studies. However, this basic assumption is not met if there is heterogeneity between studies. There are many causes of this heterogeneity such as chance, difference in cut-off value, difference in study design, prevalence, research environment, and the demographic factors of the sample population [3].

The DTA presents various methods for diagnosing the heterogeneity [3].

First, the asymmetry of the SROC curve may be a cause of heterogeneity.

Second, heterogeneity may be suspected if the degree of scattering or variation of individual studies in the SROC curve is large.

Third, heterogeneity may be suspected if the between-study variation is greater than the within-study variation in the forest plot (sensitivity, specificity, DOR).

Fourth, heterogeneity may be suspected if the correlation coefficient of sensitivity and specificity is larger than zero.

The first to third factors only depend on visual distinction, so only the overall outline can be seen.

The symmetry of the SROC curve indicates the agreement of the models of the divided SROC curves when the SROC curve is divided by a random line from the top of the y-axis to the right bottom of the x-axis. In other words, when the SROC curve is symmetrical and the inflection point is drawn to the top left corner and sharply turned, the area AUC of the SROC curve increases and the Youden’s J index (J=sensitivity+specificity-1) becomes high, which indicate a good DTA.

In visual verification, the SROC curve in this example does not appear to have a high symmetry, and the degree of scattering of individual studies also does not appear to be large.

According to the within-study and between-study variation in the forest plot (Figure 4), the between-study variation does not appear to be large.

■ Sensitivity and specificity correlation coefficient
Finally, to examine the correlation coefficient of sensitivity and specificity, additional variables are created for the current data as follows:
· dta_shim$sn <- dta_shim$TP/(dta_shim$TP+dta_shim$FN)
·dta_shim$sp <- dta_shim$TN/(dta_shim$FP+dta_shim$TN)
·dta_shim$logitsn <- log(dta_shim$sn/(1-dta_shim$sn))
·dta_shim$logitsp <- log(dta_shim$sp/(1-dta_shim$sp))

First, the sensitivity (dta_shim$sn) and specificity of each study are determined using the equations. Then, the sensitivity and specificity, which are proportion data, are logit-transformed to meet the distribution assumption. Then, the variables are checked to determine whether they have been created properly.

·View(dta_shim)

Once the variables are logit-transformed, the correlation coefficient of the sensitivity and specificity is obtained as follows:

·cor(dta_shim$logitsn, dta_shim$logitsp)

The correlation coefficient function is “cor”. When the logit-transformed sensitivity and specificity are entered in this function, a correlation coefficient of -0.227 is obtained.

If the sensitivity and specificity are mutually equal and have a normal symmetric distribution, they show a trade-off relationship. The two are balanced against each other, and when one of them is lowered, the other one is raised. Therefore, the sizes of these two measurements differ in opposite directions depending on the cut-off value in the diagnostic test, and hence, these two values inevitably have a negative correlation.

The correlation coefficient in this example is a negative value, indicating a low heterogeneity.

■ Meta regression analysis
The “mada” package does not provide functions for the meta regression analysis of the DTA. Therefore, the statistical significance of the moderating variable subgroup (Western European countries vs. other countries) is verified by performing meta regression analysis with the DOR as the effect size.
·library(meta)
·metareg(DOR_model, g, method.tau=“REML,” digits=3)

Load the “meta” package into the memory again because it was unloaded before the “mada” package was loaded.

Then, enter the DOR meta-analysis model (DOR_model) and the moderating variable g into the meta regression analysis function metareg. Next, determine the between-study variation of restricted maximum-likelihood estimator, and check the value to only three decimal places.

The meta regression analysis result confirmed that the p-value of the moderating variable g was 0.922, indicating statistical insignificance.

## CONCLUSION

This study summarized statistical theory and focused on the actual performance of meta-analysis so that it is easily understandable to general researchers who do not have majors in statistics. In other words, this study aimed to allow general researchers to adequately use already developed statistical methods in their respective fields of study to interpret the results.

Performing an analysis to determine the DTA in R software can be a complex task because one needs to use various packages. Therefore, we recommend that researchers learn the analysis method using STATA and Meta-DiSc applications as well, which can be operated as a single package.

Researchers who desire to perform an analysis of the DTA should establish the concepts of summary statistics and summary line.

We hope that this study will help domestic researchers perform meta-analysis more easily, and that it will encourage related research.

## Figures and Tables

**Table 1.** Diagnostic test accuracy summary statistics [2]

Summary statistics	Equation	Definition
Sn	TP/(TP+FN)	Proportion of persons who have positive test results to those with disease
Sp	TN/(FP+TN)	Proportion of persons who have negative test result to those without disease
PPV	TP/(TP+FP)	Proportion of persons with disease to those who have positive test result
NPV	TN/(FN+TN)	Proportion of persons without disease to those who have negative test result
LR+	Sn/(1-Sp)	Ratio of the probability of a positive test result among those with disease to that of a positive test result among those without disease
LR-	(1-Sn)/Sp	Ratio of the probability of a negative test result among those with disease to that of a negative test result among those without disease
Accuracy of index test	(TP+TN)/(TP+FP+FN+TN)	The proportion of persons who are true positive and persons who are true negative among all subjects
DOR	(TP*TN)/(FP*FN)	The ratio of the OR for a positive test result among persons with disease to that among persons without disease

Sn, sensitivity; Sp, specificity; PPV, positive predictive value; NPV, negative predictive value; LR+, positive likelihood ratio; LR-, negative likelihood ratio; DOR, diagnostic odds ratio; TP, true positive; FP, false positive; FN, false negative; TN, true negative; OR, odds ratio.

**Table 2.** Sample data for diagnostic test accuracy [2]

Id	TP	FP	FN	TN	g
Wiegmann	21	1	9	104	1
Bouhanick	49	21	7	110	1
Schwab	24	5	3	31	1
Zelmanovitz	39	6	5	48	0
Ahn	23	9	7	41	0
Ng	12	7	2	44	0
Gansevoort	10	13	3	40	1
Incerti	82	12	7	177	0
Sampaio	99	45	21	128	0

TP, true positive; FP, false positive; FN, false negative; TN, true negative; g, subgroup.
