# Supplementary material for: Diagnostic test accuracy: application and practice using R software
Source: Epidemiol Health. 2019 Mar 28;41:e2019007. doi: 10.4178/epih.e2019007 (PMC6545496; doi:10.4178/epih.e2019007)
Supplement: Supplementary file 1 [file epih-41-e2019007-supplementary.pdf]

<Supplementary Material 1>

R 진단검사 메타분석 코드

#예제자료는 dta_shim.csv(Table 2) 임.

R의 “mada” & “meta” 패키지를 이용한 진단검사 메타분석

```
#데이터 코딩 및 불러오기
dta_shim <- read.csv("dta_shim.csv", header=TRUE)
```

▶ Descriptive statistics; Univariate analysis

```
library(meta) #“meta” 패키지 불러오기

#Sensivivity & Specificity를 metaprop를 이용해서 logit변환 계산후 환원한다.
#sm=sm="PLOGIT"(logit 변환, default), "PLN"(log변환), "PRAW"(변환없음)
#method.ci="CP"(Clopper-Pearson interval also called 'exact' binomial interval, default)
sensitivity_logit <- metaprop(dta_shim$TP, dta_shim$TP + dta_shim$FN, comb.fixed=FALSE, comb.random= TRUE, sm="PLOGIT", method.ci="CP", studlab=dta_shim$id, byvar=dta_shim$g) #logit transformed
print(sensitivity_logit, digits=3)
forest(sensitivity_logit, digits=3, rightcols=c("effect", "ci"), xlab = "Sensitivity")

specificity_logit <- metaprop(dta_shim$TN, dta_shim$TN + dta_shim$FP, comb.fixed=FALSE, comb.random= TRUE, sm="PLOGIT", method.ci="CP", studlab=dta_shim$id, byvar=dta_shim$g) #logit transformed
print(specificity_logit, digits=3)
forest(specificity_logit, digits=3, rightcols=c("effect", "ci"), xlab = "Specificity")

#DOR계산하기 random 모형의 OR과 동일함.
DOR_model <- metabin(TP,TP+FP,FN,FN+TN, sm="OR",comb.fixed=FALSE,comb.random= TRUE, method ="Inverse", id, byvar=g, data=dta_shim)
print(DOR_model) #OR값과 신뢰구간을 확인할 수 있다.
forest(DOR_model, digits=3, rightcols=c("effect", "ci"), xlab = "Diagnostic Odds Ratio"

##DTA statistics table; data.frame로 만듬##
#수동입력 개별  결과에서 찾아서 수동으로 넣어야한다.
SN <- 0.841
SN95h <- 0.882
SN95l <- 0.788
SP <- 0.861
SP95h <- 0.909
SP95l <- 0.794
AUC <- 0.906 #mada 패키지 reitsma 함수에서 나온값이다.
DOR <- 37.935
DOR95h <- 79.132
DOR95l <- 18.185

#요약추정치 테이블 생성
```

```
DTA_statistics_table <- data.frame(
   "Item" = c("Sensitivity", "Specificity", "DOR", "AUC"),
   "Est" = c(SN, SP, round(DOR,3), AUC),
   "CI95l" = c(SN95l,SP95l, round(DOR95l,3),"-"),
   "CI95h" = c(SN95h,SP95h, round(DOR95h,3),"-")
)
```

▶ Descriptive statistics; bivariate analysis

```
#패키지 "meta"와 "mada"가 forest 함수를 서로 겹치게 되므로 사용하지 않는 "mada" 패키지는 일단 메모리에서 내린다.
detach(package:meta)
library(mada) # mada 패키지 불러오기

#reitsma model
fit <- reitsma(dta_shim, correction.control = "single") #reitsma모델(bivariate모델)을 fit으로 설정한다.
summary(fit) #sn만 확인가능.

#SROC plot
plot(fit, sroclwd = 2, xlim = c(0,1), ylim = c(0,1),
      main = "SROC curve (bivariate model) for Diagnostic Test Accuracy")
#sroc곡선에 개별 연구를 넣어준다.
points(fpr(dta_shim), sens(dta_shim), pch = 2)
#sroc곡선 좌하단과 우하단에 요약통계치 넣어준다. pch에 1000은 모양을 표시안하려는 속임수이다.
legend("bottomright", c("data", "summary estimate", "AUC=0.906", "DOR=37.935", "Sensivivity=0.841", "Specificity=0.861"), pch = c(20,1,1000,1000,1000,1000) )
legend("bottomleft", c("SROC", "95% CI region"), lwd = c(2,1))


##진단검사 결과의 이질성 검토##

# sensitivity 와 specificity의 상관관계;이질성 확인
dta_shim$sn <- dta_shim$TP/(dta_shim$TP+dta_shim$FN)
dta_shim$sp <- dta_shim$TN/(dta_shim$FP+dta_shim$TN)
dta_shim$logitsn <- log(dta_shim$sn/(1-dta_shim$sn))
dta_shim$logitsp <- log(dta_shim$sp/(1-dta_shim$sp))

#상관계수 구하기
cor(dta_shim$logitsn, dta_shim$logitsp)

###meta-regression##
#DOR에서 만들어놓은 DOR_model을 사용하여 메타회귀분석을 실시)
library(meta) #meta 패키지 로딩
metareg(DOR_model,g, method.tau="REML", digits=3)
```
