# Supplementary material for: Diagnostic test accuracy: application and practice using R software
Source: Epidemiol Health. 2019 Mar 28;41:e2019007. doi: 10.4178/epih.e2019007 (PMC6545496; doi:10.4178/epih.e2019007)
Supplement: Supplementary file 2 [file epih-41-e2019007-supplementary1.pdf]

Volume: 41, Article ID: e2019007

https://doi.org/10.4178/epih.e2019007

<방법론>

진단검사 메타분석: R을 활용한 적용과 실제

Diagnostic Test Accuracy: Application and Practice using R software

심 성 률[1,2], 김 성 장[3,4,5], 이 종 후[6]

[1]고려대학교 의과대학 예방의학교실, [2]순천향대학교 서울병원 비뇨의과학연구소,
[3]부산대학교 의과대학 핵의학과, [4]양산부산대학교병원, 의생명융합연구소,
[5]양산부산대학교병원, 핵의학과, [6]제주대학교 의과대학 내과

교신저자: 심성률

sungryul.shim@gmail.com

서울시 성북구 안암로 145

.

Volume: 41, Article ID: e2019007

https://doi.org/10.4178/epih.e2019007

Abstract

The objective of this study was to describe general approaches of diagnostic test accuracy that are available for quantitative synthesis of data using R software.

We conducted a diagnostic test accuracy that summarized statistics for univariate analysis and bivariate analysis. The package commands of R software were metaprop and metabin for sensitivity, specificity, and diagnostic odds ratio, forest for forest plot, reitsma of mada for summarized ROC curve, and metareg for meta-regression analysis. The estimated overall effect sizes, test for heterogeneity and moderator effect, and a summarized ROC curve were reported using R software. Especially authors stressed how to calculate effect sizes of target studies in diagnostic test accuracy. This study focused on the practical methods of diagnostic test accuracy rather than theoretical concepts for Korean researchers who were non-majored in statistics. Through this study, authors hope that many Korean researchers will use R software to perform a diagnostic test accuracy more easily and that related research will be activated.

Keywords: Meta-analysis, Diagnostic test accuracy, Receiver-operating characteristic curve, Likelihood ratios, Nomograms, mada, reitsma.

Volume: 41, Article ID: e2019007

https://doi.org/10.4178/epih.e2019007

서론

일반적인 메타분석(pairwise meta-analysis)에서는 하나의 효과크기(effect size)를 산출하는데 예를 들어 이분형 자료는 상대위험도(relative risk, RR)와 오즈비(odds ratio, OR)를 그리고 연속형 자료는 평균의 차이(mean difference) 등이 있다.

반면에 진단검사 메타분석(diagnostic test accuracy)에서는 동시에 두 가지 효과크기를 종합하는데 예를 들어 민감도(sensitivity)와 특이도(specificity) 또는 양성예측도(Positive Predictive Value)와 음성예측도(Negative Predictive Value) 등이 있다 [1-3].

따라서 진단검사 메타분석은 결과값이 하나인 일반적인 메타분석보다 복잡해진다.

결국 결과값이 두개 이상인 다변량 분석으로의 확장은 다층개념의 도입이 불가피하며 이를 위해서 어느정도의 수리학적 이해와 통계 프로그램의 운용 능력이 요구되어 진다.

본 연구는 진단검사 메타분석을 위해서 반드시 이해해야 할 요약추정치를 만들어내는 개념과 더불어 R 소프트웨어 실행방법에 초점을 맞추었다.

본 연구는 저자가 수행했던 메타분석 선행연구들을 [1-3] R 소프트웨어를 이용해서 다시 풀어낸 것이다. 또한 본 연구는 진단검사 요약추정치 계산을 위한 효과크기의 유형과 변경부터 다룰 것이기 때문에 진단검사 메타분석에 대한 사전 지식이 수반되어야 한다.

1. 진단검사 메타분석의 이해

일반적으로 진단검사의 데이터는 2X2 테이블 형태를 띄며 가로는 검사 유/무, 세로는 질병의 유/무로 구분한다 (Figure 1).

Volume: 41, Article ID: e2019007

https://doi.org/10.4178/epih.e2019007

1.1. 진단검사 요약추정치

진단검사 메타분석은 진양성(true positive, TP), 위양성(false positive, FP), 위음성(false negative, FN), 진음성(true negative, TN)의 4가지 기초정보로부터의 요약결과인 요약추정치(summary statistics)와 요약곡선(summary line)으로 보여주게 된다. 대표적인 요약추정치에는 통합된 민감도(sensitivity), 특이도(specificity), 진단오즈비(DOR, diagnostic odds ratio), 그리고 forest plot이 있으며 요약곡선은 SROC(summary receiver operating characteristic) 곡선이 있다 (Table 1).

1.2. 진단검사 메타분석 모형

진단검사 메타분석에서의 요약추정치를 계산하기 위해서는 일반적인 메타분석과 마찬가지로 적합한 모형이 선택되어져야 하며 민감도와 특이도를 동시에 고려한 모형으로 Moses-Littenberg SROC 모형 [4,5], Bivariate 모형 [6], 그리고 Hierarchical SROC [7] 일반적으로 활용되고 있다.

Moses-Littenberg 모형은 진단검사 메타분석을 위해 초기에 만들어진 간단한 모델로서 단순회귀분석(simple linear regression)으로 SROC를 추정한다. 이는 일반 메타분석에서의 고정효과(fixed effect) 모형과 유사한 것으로 연구들간의 이질성(heterogeneity)을 추정할 수 없다. 또한 전체 변동 중 연구 내 변동과 연구 간 변동을 분리할 수 없는 모형으로 모수 추정치와 표준편차, 신뢰구간을 제공하지 않고 SROC 곡선만을 구할 수 있어 분석이 제한적이다.

Moses-Littenberg 모형의 단점을 극복하고자 위계적 모형(hierarchical model)을 바탕으로 개발된 것이 Bivariate 모형과 HSROC(Hierarchical SROC) 모형으로 공변량이 없을 때 수리적으로 두 모형은 동일한 값을 제공한다 [8,9]. 일반 메타분석에서의 변량효과(random effect) 모형과 유사하며 두 모형 모두 연구들의 연구내 변동과 연구간 변동을 추정할 수 있다. 즉, 이질성을 추정할 수 있는 것이다.

Bivariate 모형은 연구 내 변동은 민감도와 특이도를 직접 모형화한 이항분포(binominal distribution)를 가정하고 연구 간 변동은 이변량 정규분포(bivariate normal distribution)를 가정하는 반면 HSROC 모형은 연구 내 변동은 이항분포를 가정하고 연구 간 변동은 이항분포의 확률에 대해 로지스틱 회귀모형을 적용하여 로지스틱 모형에 포함된 모수들에 대해 위계적 분포를 가정한다는 것이 다른 점이라고 할 수 있다.

본서에서 실습할 R “mada” 패키지 reitsma 모델은 기본적으로 Bivariate 모형으로 요약추정치를 산정하고 SROC 곡선을 추정한다.

1.3. 효과크기 계산

Table 1의 민감도와 특이도를 생각해보자.

민감도는 TP/(TP+FN)이며 특이도는 TN/(TN+FP)로서 비율의 형태이다.

이러한 비율형 자료는 원자료 형태보다는 로짓변환한 자료를 많이 활용하고 있는데 이것은 자료의 분포를 통계의 가정(assumption)에 맞추기 위한 방법이다.

비율형 자료는 0과 1사이로 상·하한이 닫혀 있어 처음에 승산변환을 해서 상한을 풀어주고 이어서 로그변환을 해서 하한을 풀어주어야 자료의 분포가 통계의 가정에

적합한 자료의 형태가 되며 이를 로짓변환이라고 한다.

물론 진단검사 요약추정치의 계산이 완료되고 나면 해석을 위해서 이를 처음의 수치로 환원시킨다. R을 이용한 실습에서 “meta” 패키지의 metaprop 함수를 사용하면서 로짓변환한 민감도와 특이도를 계산한 후 환원시켜서 해석할 것이므로 효과크기를 왜 변환했는지를 잘 이해해야 할 것이다.

R의 “mada” & “meta” 패키지를 이용한 진단검사 메타분석

Figure 2는 진단검사 메타분석의 흐름을 나타낸다. 최초 자료 코딩시 해당 함수에 적합하도록 변수명을 수정해야하며 메타분석 모델선정(Fixed or Random)으로 종합 효과크기를 제시하고 -> 이질성을 확인한 다음 -> 출판 편향을 확인해서 보고한다.

R에서 진단검사 메타분석을 실행하려면 "mada" 패키지가 필요한데 “mada”를 설치하고 나면 "mvtnorm", "ellipse", "mvmeta"도 필요하다고 나오니 미리 관련 패키지를 설치한다.

```
·install.packages("mada")
·install.packages("mvtnorm")
·install.packages("ellipse")
·install.packages("mvmeta")
```

또한 R에서 일반 중재 메타분석을 실시하는 패키지 “meta”, “metafor” 그리고 “rmeta”도 미리 설치해 둔다.

```
·install.packages("meta")
·install.packages("metafor")
·install.packaqes("rmeta”)
```

주요 설명은 “mada”와 “meta” 패키지를 중심으로 기술한다. “mada” 패키지에 대한 상세한 설명은 각 패키지별 상세 코드, 자료, 참고문헌 등이 있으니 참고하기 바란다 [10].

Volume: 41, Article ID: e2019007

https://doi.org/10.4178/epih.e2019007

본문과의 구분을 위하여 명령어 앞에는 '·'으로 표시하였다. 명령어가 길어져서 다음 줄로 넘어가더라도 '·' 없으면 앞의 줄에서 이어지는 것이다. 따라서 실제 R 프로그램에 입력시에는 '·'는 제외하고 타이핑하여야 한다.

1. 데이터 코딩 및 불러오기

진단검사 메타분석을 위한 예제로서 (Table 2) 당뇨환자에 있어서 미세단백뇨 (microalbuminuria) 확인을 위한 검사법 중 UAC(urine sample measuring the albumin concentration) 방법을 발췌한 것이다 [2,3,11]. TP, FP, FN, TN은 각각 진양성, 위양성, 위음성, 진음성을 나타낸다. subgroup (g) 1은 서구유럽국가들이며 0은 서구유럽국가를 제외한 나라로 구분하였다.

작업폴더에 넣어둔 예제 파일을 아래의 명령어로 R의 메모리에 불러온다. 한가지 주의할 점은 R에서는 쉼표로 구분된 수치파일(csv)의 형태를 선호하니 Table 2를 "dta_shim.csv" 포멧으로 저장해서 지정된 작업폴더에 넣어 두어야한다.

```
·dta_shim <- read.csv("dta_shim.csv", header=TRUE)
```

read.csv는 csv파일을 불러오는 함수로서 파일명 "dta_shim.csv"를 불러와서 파일의 첫번째 변수명을 그대로 쓴다는 뜻이다 (header=TRUE). 이렇게 로딩된 파일은 R 메모리에서는 dta_shim 이름의 데이터로 저장된다. 이를 확인해보려면 View 함수에 지정한 데이터를 넣어준다.

2. 요약추정치

진단검사 메타분석 요약추정치 계산을 위한 이변량 분석모델 (bivariate model)인

Volume: 41, Article ID: e2019007

https://doi.org/10.4178/epih.e2019007

“mada” 패키지에서는 요약추정치의 - 민감도(sensitivity), 특이도(specificity), 그리고 진단오즈비(diagnostic odds ratio) - 종합 효과크기를 제시하지 않고 있으며 단순히 개별 연구들의 효과크기만을 forest plot을 보여주고 있어서 불편하다.
따라서 “meta” 패키지를 활용한 단변량 분석을 먼저 실시하여 각각의 요약추정치 값을 확인한 이후 “mada”패키지를 사용하여 SROC 곡선을 제시하는 것이 자연스럽다.

1) 단변량 분석 (Univariate analysis)

민감도(sensitivity), 특이도(specificity), 그리고 진단오즈비(diagnostic odds ratio)를 단변량 분석모델로 계산하고 이를 도식화한다.

메타분석을 실행하기위해 meta 패키지를 로딩시킨다.

```
·library(meta)
```

(1) 민감도 (Sensitivity)

“meta” 패키지는 하위에 여러 함수들을 포함하는데 그중 metaprop 함수는 비율형 자료에서 사건수(event)과 표본수(n)이 있을 때 종합효과크기를 계산한다.

```
·sensitivity_logit <- metaprop(dta_shim$TP, dta_shim$TP + dta_shim$FN, comb.fixed=FALSE,
  comb.random= TRUE, sm="PLOGIT", method.ci="CP", studlab=dta_shim$id,
  byvar=dta_shim$g)
·print(sensitivity_logit, digits=3)
```

민감도 분석에서의 사건발생수는 TP이며 표본수는 TP + FN이다. R에서는 해당 데이터의 변수를 사용하기 위해서는 ‘$’ 표시를 사용한다 (예: dta_shim 데이터의 TP 변

수를 지정한다면 dta_shim$TP). metaprop 함수에 사건수(dta_shim$TP)와 표본수 (dta_shim$TP+dta_shim$FN)을 차례대로 입력한 후 뒤로는 옵션들을 위치시킨다.

비율형 자료에서 효과크기를 계산하는 방식 중 로짓변환 후 환원하는 계산방법을 사용하였다. 이외에도 자료를 변환하지 않은 원자료를 그대로 사용하고자 한다면 sm="PRAW"를 입력하고, 로그변환 후 환원한 결과값을 알고 싶다면 sm="PLN"로 설정한다.

통계모형의 가정에 충실하고 자료의 대칭성과 분포를 감안했을 때 비율형 자료는 변환(로그변환 또는 로짓변환)하는 것이 보수적인 결과를 만들어내어 보다 바람직하다. 그러나 많은 선행연구와 통계모형들이 연구자의 운용범위를 폭넓게 열어놓았기에 자신의 연구결과에 적합한 방법을 찾아서 잘 활용하면 된다.

자료변환을 실시하더라도 metaprop 함수가 자동으로 환원해서 해석할 수 있는 종합효과크기를 표시한다.

아울러 신뢰구간을 계산하는 방법도 여러가지가 있는데 이건 너무 복잡하지않게 default로 주어지는 Clopper-Pearson 방법을 사용하자 (method.ci="CP").

Random effect model를 사용하였으며 comb.fixed=FALSE, comb.random= TRUE를 추가해서 입력한다. 원하는 모형을 FALSE 또는 TRUE에 따라 선택 가능하다.

Studlab=study는 개별 연구들의 이름을 나타내며 subgroup별 결과를 나타내려면 byvar=g를 입력하는데 g는 subgroup을 나타내는 변수명이다. metaprop 함수를 사용해서 나온 결과들은 sensitivity_logit에 지정되며 결과는 다음과 같다 (Figure 3).

sensitivity_logit에서 나온 결과들을 Figure 3을 하나씩 살펴보자.

Volume: 41, Article ID: e2019007

https://doi.org/10.4178/epih.e2019007

①은 전체 9개 연구의 종합 효과크기를 나타낸다. Random effect model의 proportion은 0.841 (95%CI; 0.788, 0.882)을 나타내었다.

②은 subgroup에 해당하는 결과를 나타낸 것이다. Random 모델에서는 subgroup(0 vs 1)에 따라 민감도의 차이가 약간 보여진다. Random effect model을 기준으로 서구유럽국가들은 0.816, 그외 국가에서는 0.855로서 추후 국가별 메타회귀분석을 실시해서 이를 검정하여야 한다.

③는 전체 연구의 이질성(heterogeneity)를 나타낸 것이다. 이질성의 Higgins'I^2는 Cochrane Q statistics에서 자유도(degree of freedom)를 뺀 것을 다시 Cochrane Q statistics으로 나누어 준 값으로 이질성을 일관성 있게 정량화시킨다. 0%에서 40%는 이질성이 중요하지 않을 수 있으며 (might not be important), 30%에서 60%는 중간 이질성(moderate heterogeneity), 50%에서 90%는 중대한 이질성(substantial heterogeneity), 그리고 75%에서 100%는 심각한 이질성(considerable heterogeneity)을 나타낸다. Cochrane Q statistics의 *p*-value는 조금 폭 넓게 0.1을 유의성 판단 기준으로 한다 [3].

본 민감도 분석에서의 Higgins'I^2는 32.5%이며 Cochrane Q statistics *p*-value = 0.158로서 이질성이 약하다는 것을 알 수 있다.

그 외 Figure 3 하단에 해당 결과가 어떤 계산방법으로 도출되었는지 밝히고 있다. Inverse variance method는 메타분석의 기본적인 방법으로서 개별 연구들의 가중치를 계산할 때 해당 연구의 역분산을 활용한다. DerSimonian-Lair estimator는 Random effect model에서 연구간 변량을 계산할 때 tau값을 계산하였다는 것이다. Logit transformation(로짓 자료 변환을 실시하였음)과 Clopper-Pearson 방법을 사용하였다.

Volume: 41, Article ID: e2019007

https://doi.org/10.4178/epih.e2019007

■ Forest plot(숲 그림)

·forest(sensitivity_logit, digits=3, rightcols=c("effect", "ci"), xlab = "Sensitivity")

forest 함수에 해당 설정된 메타분석 모델(sensitivity_logit)을 입력한다. 그런 다음 다양한 옵션들을 넣어주어 식별이 용이하게 만들어준다. digits=3은 소수점 세자리까지만 표시, rightcols=c(“effect”,”ci”))는 forest plot 오른편에는 원래는 weight가 표시되는데 이를 생략하고 효과크기와 신뢰구간만을 보여주라는 뜻이다.

이외에도 색깔을 넣거나 필요한 정보를 추가/제거하는 등 임의대로 작성할 수 있으니 보다 상세한 내용은 직접 meta 패키지를 연습해보기 바란다.

Forest plot은 앞의 종합 효과크기와 동일한 정보를 제공한다. 더불어 개별연구들의 효과크기를 그래픽으로 제시함으로써 연구 내 변동과 연구 간 변동을 쉽게 파악할 수 있도록 해준다.

예를 들어 연구 내 변량이 큰 것은 Gansevoort, Ng, Wiegmann 그리고 Ahn인 것을 알 수 있고 연구 간 변량이 큰 것은 Wiegmann과 Incerti인 것을 알 수 있다.

(2) 특이도 (Specificity)

·specificity_logit <- metaprop(dta_shimTN, dta_shimTN + dta_shim$FP, comb.fixed=FALSE, comb.random= TRUE, sm="PLOGIT", method.ci="CP", studlab=dta_shim$id, byvar=dta_shim$g)
·print(specificity_logit, digits=3)

특이도 분석에서의 사건발생수는 TN이며 표본수는 TN + FP이다. metaprop 함수에 사건수(dta_shim$TN)와 표본수(dta_shim$TN+dta_shim$FP)을 차례대로 입력한 후 뒤로는 옵션들을 위치시킨다. 이하 설명은 민감도 분석과 동일하다.

Volume: 41, Article ID: e2019007

https://doi.org/10.4178/epih.e2019007

specificity_logit에서 나온 결과들을 하나씩 살펴보자.

전체 9개 연구의 종합 효과크기를 나타낸다. Random effect model의 proportion은 0.861 (95%CI; 0.794, 0.909)을 나타내었다.

Random 모델에서는 subgroup(0 vs 1)에 따라 특이도의 차이가 거의 보이지 않는다. 본 특이도 분석에서의 Higgins' I^2는 78.3%이며 Cochrane Q statistics *p*-value <0.0001로서 이질성이 존재한다는 것을 알 수 있다.

■ Forest plot(숲 그림)

```
·forest(specificity_logit, digits=3, rightcols=c("effect", "ci"), xlab = "Specificity")
```

이하 설명은 민감도 분석과 동일하다.

(3) 진단오즈비 (diagnostic odds ratio)

“meta” 패키지는 하위에 여러 함수들을 포함하는데 그중 metabin 함수는 이분형 자료에서 원자료들이 모두 있을 때 종합효과크기를 계산한다. 민감도와 특이도를 개별로 구할 때는 비율형 자료에 해당하지만 2x2 형태의 진단오즈비(DOR)은 이분형 자료이다.

```
·DOR_model <- metabin(TP,TP+FP,FN,FN+TN, sm="OR",comb.fixed=FALSE,comb.random=
  TRUE, method ="Inverse", id, byvar=g, data=dta_shim)
·print(DOR_model)
```

패키지 상세설명을 하나씩 훑어보면 이해가 갈 것이지만 빠른 이해를 위해서 가급적 변수명은 본 예제에서 변경하지 않도록 한다.

이분형 자료에서는 TP, TP+FP, FN, FN+TN을 각각 차례대로 넣어준다.

Volume: 41, Article ID: e2019007

https://doi.org/10.4178/epih.e2019007

효과크기를 OR 표시하고 개별 연구들의 가중치는 일반적인 inverse variance method를 사용한다 (method="Inverse").

연구간 변량을 고려한 Random effect model의 설정을 위해서 comb.fixed= FALSE, comb.random=TRUE를 추가해서 입력한다.

id는 개별 연구들의 이름을 나타내며 data=dta_shim은 R 메모리에 로딩된 dta_shim이라는 데이터를 지정해주는 것이다. subgroup별 결과를 나타내려면 byvar=g를 입력하는데 g는 subgroup을 나타내는 변수명이다. metabin 함수를 사용해서 나온 결과들은 DOR_model에 지정된다.

■ Forest plot(숲 그림)

·forest(DOR_model, digits=3, rightcols=c("effect", "ci"), xlab = "Diagnostic Odds Ratio")

DOR_model에서 나온 결과들을 Figure 4에서 하나씩 살펴보자.

전체 9개 연구의 종합 효과크기를 나타낸다. Random effect model의 OR은 37.935 (95%CI; 18.186, 79.132) p-value <0.0001로서 해당 진단검사를 통해서 질병이 없는 사람 중 검사결과가 양성일 오즈비에 비해 질병이 있는 사람 중 검사결과가 양성일 오즈비가 약 38배 높게 나타났다.

Random 모델에서는 subgroup(0 vs 1)에 따른 차이가 거의 없는 것으로 보여진다.

전체 연구의 이질성(heterogeneity)은 Higgins' I^2는 72.7%이며 Cochrane Q statistics p-value = 0.0003로서 이질성이 존재한다는 것을 알 수 있다.

2) 이변량 분석 (Bivariate analysis)

Volume: 41, Article ID: e2019007

https://doi.org/10.4178/epih.e2019007

이변량 분석을 위한 “mada” 패키지는 다른 진단검사 메타분석 프로그램인 Meta-DiSc 또는 STATA 에서와 같이 민감도와 특이도 그리고 진단오즈비를 직접 제시하지 않는다. 심지어 forest plot 조차도 종합 효과크기는 없고 단순히 개별 연구들의 효과크기만을 보여주고 있어 종합된 결과를 보여주려면 source code를 찾아서 이를 계산해주어야 한다.

따라서 본 연구에서는 단변량 분석에서 이미 요약추정치를 – 민감도, 특이도, 진단오즈비 –각각 실행하였으며 이어지는 이변량 분석에서는 “mada” 패키지를 활용하여 SROC 곡선만을 추정하고자 한다.

“mada” 패키지를 로딩하기에 앞서서 앞에서 사용한 “meta”패키지를 내려준다. 왜냐하면 “mada”와 “meta”는 forest 함수를 같이 사용하는데 이것이 겹쳐져서 실행이 안될 수 있기 때문이다.

```
·detach(package:meta)
```

(1) 진단검사 메타분석 요약곡선 (SROC 곡선)

이변량 분석을 위한 패키지 로딩시킨다.

```
·library(mada)
```

“mada” 패키지를 이용해서 민감도, 특이도, 진단오즈비의 단변량 분석 그림을 보고자하면 다음과 같이 입력한다.

```
·forest(madad(dta_shim), type="sens", xlab = "Sensitivity", snames = dta_shim$id)
·forest(madad(dta_shim), type="spec", xlab = "Specificity", snames = dta_shim$id)
·forest(madauni(dta_shim))
```

앞에서 단변량 분석에서 구하였던 그림과 동일하며 요약추정치의 종합효과크기를

Volume: 41, Article ID: e2019007

https://doi.org/10.4178/epih.e2019007

제시하지 않아 추천하지 않는다.

“mada” 패키지 중에서 이변량 모델에 적합한 reitsma 함수를 사용한다.

·fit <- reitsma(dta_shim, correction.control = "single")
·summary(fit)

reitsma 함수에 dta_shim 데이터를 넣고, 데이터의 셀에 '0'일 경우 계산불능 방지를 위해서 전체 연구의 모든 셀에 0.5를 넣거나 (correction.control = "all") 또는 해당연구(가로)의 셀들만 보정 (correction.control = "single")할 수 있다. 옵션에서 ‘correction = 0.5’ 처럼 임의 수치로 조절할 수 있고, 0.5가 default이다. reitsma 함수를 사용한 모델은 fit으로 지정된다.

콘솔 창 중간 부분에 AUC값이 0.906을 표시하고 있으며 아울러 HSROC 모델에 해당하는 값들도 표시되니 참고하기 바란다.

본격적으로 SROC 곡선을 그려보자 (Figure 5). 그래프를 명령어 순서대로 그릴것이며 메모리상에 제일 처음 그린 SROC 곡선이 남아 있으므로 겹쳐져서 만들어진다.

·plot(fit, sroclwd = 2, xlim = c(0,1), ylim = c(0,1), main = "SROC curve (bivariate model) for Diagnostic Test Accuracy")

plot은 그래프를 그리는 함수이다. 설정된 모델 fit을 넣고 sroclwd=2는 sroc곡선의 두께를 나타내며, xlim과 ylim을 조절해서 x,y축의 단위를 조절한다. 현재는 최소 0에서 최대 1까지를 표시한 것이다.

·points(fpr(dta_shim), sens(dta_shim), pch = 2)

points는 개별 연구를 넣어준다. fpr()과 sens()는 각각 해당 데이터내의 개별 연구의 false positive rate와 sensitivity를 표시하는 것이며 pch=2은 세모 모양을 나타낸다. 네모(0), 동그라미(1), 세모(2), 십자가(3), 가위표(4), 마름모(5), 역삼각형(6), 별표(8),

Volume: 41, Article ID: e2019007

https://doi.org/10.4178/epih.e2019007

검은점(20) 등 다양하며 검은점(20)이 가장 식별력이 좋아보인다 (Figure 5).

·legend("bottomleft", c("SROC", "95% CI region"), lwd = c(2,1))
SROC 곡선의 좌하단에 곡선의 설명을 위한 주석이다.

(2) 이질성 검토

요약추정치와 SROC 요약곡선을 제시하면 진단검사 메타분석의 주요부분은 구해진 것이다. 이후의 과정은 일반 메타분석과 마찬가지로 연구의 이질성을 확인하고 만약 유의한 인자가 있다면 이를 검정하고 보고하여야 한다.

SROC 곡선의 기본 가정은 모든 연구에서 ROC 곡선의 형태가 동일하다는 것이다. 그렇지만, 연구들 간의 이질성이 존재한다면 이러한 기본 가정을 충족하지 못한다. 이러한 이질성의 원인은 우연(chance), cut-off value 차이, 연구설계 차이, 유병률, 연구환경, 그리고 표본집단의 인구사회학적 요인에 이르기까지 매우 다양하다 [3].

진단검사 메타분석에서는 이런 이질성의 여부를 진단할 수 있는 다양한 방법들을 제시하고 있다 [3].
첫째, SROC 곡선의 비대칭성이다.
둘째, SROC 곡선내 개별 연구들의 흩어짐 정도이다. 연구들의 변량이 크다면 이질성을 의심할 수 있다.
셋째, Forest plot(민감도, 특이도, 진단오즈비)에서 연구 간 변동이 연구 내 변동보다 큰 경우 이질성을 의심할 수 있다.
넷째, 민감도와 특이도의 상관계수가 0보다 큰 양수인 경우 이질성을 의심할 수 있다.

Volume: 41, Article ID: e2019007

https://doi.org/10.4178/epih.e2019007

첫번째부터 세번째까지 모두 시각적인 구분에만 의존하기에 전체적인 윤곽만을 파악할 수 있다.

SROC 곡선의 대칭성이란 SROC 곡선의 Y축 상단에서 X축 우하단으로 임의의 직선을 나누었을 때 양분되어진 SROC 곡선의 모형이 일치하는 지를 보는 것이다. 즉, SROC 곡선이 대칭성을 나타내며 변곡점이 좌측 상단으로 그려지며 급격히 꺽힘으로서 SROC 곡선 아래면적(AUC, area under the curve)이 커지게 되고 Youden's J index(J = 민감도 + 특이도 -1)가 높아져 좋은 진단검사임을 가늠할 수 있다.
본 예제의 SROC 곡선은 육안으로 보기에 비대칭성은 크지 않을 것으로 판단되며 개별 연구들의 흩어짐의 정도도 크지 않아 보인다.

Forest plot (Figure 4)에서의 연구간 변량과 연구내 변량을 살펴보면 연구간 변량이 크다고 판단되지 않는다.

■ 민감도 특이도 상관계수

마지막으로 민감도와 특이도의 상관계수를 알아보기 위하여 현재의 데이터에 변수를 추가로 만든다.

```
·dta_shim$sn <- dta_shim$TP/(dta_shim$TP+dta_shim$FN)
·dta_shim$sp <- dta_shim$TN/(dta_shim$FP+dta_shim$TN)
·dta_shim$logitsn <- log(dta_shim$sn/(1-dta_shim$sn))
·dta_shim$logitsp <- log(dta_shim$sp/(1-dta_shim$sp))
```

우선 수식을 이용하여 개별 연구별 민감도(dta_shim$sn)와 특이도를 구한다. 그런 다음 민감도와 특이도는 비율 자료로서 분포의 가정을 만족시키기 위하여 각각 로짓변환 시킨다. 변수가 잘 생성되었는지 확인해본다.

```
·View(dta_shim)
```

Volume: 41, Article ID: e2019007

https://doi.org/10.4178/epih.e2019007

로짓변환된 변수가 생성되어졌으면 이제 민감도와 특이도의 상관계수를 구한다.

·cor(dta_shim$logitsn, dta_shim$logitsp)

상관계수를 구하는 함수는 cor이며 로짓변환된 민감도와 특이도를 넣어주면 -0.227의 상관계수를 구할 수 있다.

민감도와 특이도가 상호 균등하고 정상적인 대칭 분포이라면 상충관계(trade-off)를 보이는 데 이것은 서로 대립하면서 균형을 맞추게 되어 한쪽이 낮아지면 반대쪽이 올라가게 되어있다. 따라서 특정 진단검사에서 cut-off value를 어떻게 설정하느냐에 따라서 두 측정치의 크기는 서로 반대방향으로 달라지게 되며 따라서 두 측정치의 상관관계는 음의 값을 가질 수밖에 없다.

본 예제의 상관계수는 음의 값을 나타냄으로 이질성이 높지 않을 것으로 판단된다.

■ 메타회귀분석

“mada”패키지에서 진단검사 메타회귀분석을 위한 기능을 제공하지 않는다. 따라서 진단오즈비를 효과크기로 하는 메타회귀분석을 실시하여 조절변수 g(서구유럽국가 vs 기타국가)에 대한 통계적 유의성을 확인한다.

·library(meta)
·metareg(DOR_model, g, method.tau="REML", digits=3)

앞에서 “mada” 패키지를 실시할 때 “meta”를 로딩 해제하였기 때문에 다시 메모리에 패키지를 로딩한다.

이후 메타회귀분석 함수 metareg에 진단오즈비 메타분석모델(DOR_model)과 조절변수로 예상되는 변수 g를 입력한다. 이후는 REML 연구간변량(tau)를 구해서 소수점 3자리까지만 확인하였다.

Volume: 41, Article ID: e2019007

https://doi.org/10.4178/epih.e2019007

메타회귀분석 결과 조절변수 g의 *p*-value = 0.922로서 통계적으로 유의하지 않는 것으로 나타났다.

맺음말

본 연구는 통계학을 전공하지 않은 일반 연구자들도 쉽게 수행할 수 있도록 통계학 이론을 최소화하고 메타분석의 실질적 수행방법에 집중하였다. 즉, 일반 연구자들이 이미 개발되어진 통계방법들을 잘 활용하여 본인의 연구분야에 적합하게 쓰고 해석하기 위함이다.

R 소프트웨어에서 진단검사 메타분석 (Diagnostic Test Accuracy)을 실시하기 위해서는 다양한 패키지를 이용해야 하기에 복잡할 수 있다. 따라서 단일 패키지로 운용할 수 있는 STATA와 Meta-DiSc 프로그램을 활용한 분석방법도 병행학습하기를 권고한다 [1-3].

진단검사 메타분석을 실행하고자 하는 연구자는 반드시 진단검사 요약 추정치와 요약곡선에 대한 개념 정립을 확고히 하여야 한다.

아울러 본 연구를 통하여 많은 국내 연구자들이 보다 쉽게 메타분석을 수행하고 관련 연구가 활성화되기를 바란다.

https://doi.org/10.4178/epih.e2019007

References

1. Hwang SD & Shim SR. Meta-analysisp; from forest plot to network meta-analysis. Seoul: Hannarae publishing co.; 2018 (Korean). ISBN: 9788955662214

2. Shim SR. Diagnostic Test Accuracy using R & Meta-DiSc software. Gyeonggi-do: SDB Lab; 2019 (Korean). ISBN 979-11-965933-2-2.

3. Shim SR, Shin IS, Bae JM. Meta-analysis of Diagnostic Tests Accuracy using STATA Software. Journal of Health Informatics and Statistics 2015; 40(3):190-199.

4. Moses LE, Shapiro D, Littenberg B. Combining independent studies of a diagnostic test into a summary ROC curve: data analytic approaches and some additional considerations. Statistics in Medicine 1993;12(14):1293-1316.

5. Littenberg B, Moses LE. Estimating diagnostic accuracy from multiple conflicting reports: a new meta analytic method. Medical Decision Making 1993;13(4):313-321.

6. Reitsma JB, Glas AS, Rutjes AW, Scholten RJ, Bossuyt PM, Zwinderman AH. Bivariate analysis of sensitivity and specificity produces informative summary measures in diagnostic reviews. Journal of Clinical Epidemiology 2005;58(10):982-990.

7. Rutter CM, Gatsonis CA. A hierarchical regression approach to meta analysis of diagnostic test accuracy evaluations. Statistics Medicine 2001;20(19):2865-2884.

8. Arends LR, Hamza TH, van Houwelingen JC, Heijenbrok-Kal MH, Hunink MG, Stijnen T. Bivariate random effects meta-analysis of ROC curves. Medical Decision Making 2008;28(5):621-638.

9. Harbord RM, Deeks JJ, Egger M, Whiting P, Sterne JA. A unification of models for metaanalysis of diagnostic accuracy studies. Biostatistics 2007;8(2):239-251.

10. R software "mada" packages. Available from: https://cran.r-project.org/web/packages/mada/index.html

11. Wu, H. Y., Peng, Y. S., Chiang, C. K., Huang, J. W.. Hung, K. Y., Wu, K. D., Tu, Y. K. & Chien, K. L. (2014). Diagnostic performance of random urine samples using albumin concentration vs ratio of albumin to creatinine for microalbuminuria screening in patients with diabetes mellitus: a systematic review and meta-analysis. JAMA Internal Medcine., 174(7), 1108-1115.
